# DIZZYNET: the European network for vertigo and balance research

**DOI:** 10.1007/s00415-015-7920-3

**Published:** 2016-04-15

**Authors:** Andreas Zwergal, Thomas Brandt, Mans Magnusson, Christopher Kennard

**Affiliations:** Department of Neurology, University of Munich, Munich, Germany; German Center for Vertigo and Balance Disorders, DSGZ, University of Munich, Munich, Germany; Clinical Neurosciences, University of Munich, Munich, Germany; Department of Otorhinolaryngology, Clinical Sciences, Lund University and Skåne University Hospital, Lund, Sweden; Nuffield Department of Clinical Neurosciences, University of Oxford, Oxford, UK

DIZZYNET, a European network for management of dizzy patients and for vertigo and balance research, was founded in October, 2014 by international experts from 13 countries all over Europe. Its structural concept, aims, and visions are presented in the supplement’s first article. The supplement documents the constitutive meeting that took place in Sonnenhausen, Bavaria last year. The other articles, which are based on expert talks presented at the meeting, reflect current developments and controversies in neuro-otology.

DIZZYNET will not compete with national or international societies active in the field. It sees its central aim in collaboration and exchange among scientists, physicians, technicians, and physiotherapists working in the field of vertigo and balance disorders. Working groups have been established on basic and translational research, clinical management, clinical trials, rehabilitation, and epidemiology in order to promote multiprofessional education. The European DIZZYNET will organize yearly international master classes on treatment of vertigo and balance disorders. The concept of this master class is “Two Days of Teaching”: how to manage dizzy patients and how to conduct otoneurological research in form of lectures for all participants and hands-on courses with an occupational focus.

